# AMPK inhibits Smad3‐mediated autoinduction of TGF‐β1 in gastric cancer cells

**DOI:** 10.1111/jcmm.16308

**Published:** 2021-02-03

**Authors:** Junrong Zou, Cong Li, Shanshan Jiang, Lingyu Luo, Xiaohua Yan, Deqiang Huang, Zhijun Luo

**Affiliations:** ^1^ Jiangxi Provincial Key Laboratory of Tumor Pathogenesis and Molecular Pathology Department of Pathophysiology School of Basic Medical Sciences Nanchang University Nanchang China; ^2^ Institute of Urology the First Affiliated Hospital of Gannan Medical University Ganzhou China; ^3^ Pharmacy Department Xiangyang Stomatological Hospital Affiliated Stomatological Hospital of Hubei University of Arts and Science Xiangyang China; ^4^ Institute of Hematological Research Shaanxi Provincial People’s Hospital Xi’an China; ^5^ Department of Gastroenterology Research Institute of Digestive Diseases the First Affiliated Hospital of Nanchang University Nanchang China; ^6^ Department of Biochemistry School of Basic Medical Sciences Nanchang University Nanchang China

**Keywords:** AMPK, Smad3, TGF‐β1

## Abstract

We have previously shown that adenine monophosphate‐activated protein kinase (AMPK) regulates transforming growth factor β (TGF‐β)‐triggered Smad3 phosphorylation. Here we report that AMPK inhibits TGF‐β1 production. First, metformin reduced mRNA levels of TGF‐β1 in gastric cancer cells, in parallel to the decrease of its protein abundance. The effects were more prominent in the cells containing LKB1, an upstream kinase of AMPK. Second, knockdown of Smad3 by siRNA abrogated the expression of TGF‐β1. Third, metformin suppressed firefly luciferase activity whose transcription was driven by TGF‐β1 promoter. In accordance, deletion of the putative binding site of Smad3 in the TGF‐β1 promoter region severely impaired the promoter activity and response to metformin. Fourth, in support of our in vitro study, clinical treatment of type 2 diabetes with metformin significantly reduced the plasma level of TGF‐β1. Finally, immunohistochemical studies revealed that TGF‐β1 was highly expressed in human gastric cancer tissues as compared with adjacent normal tissues. In contrast, p‐AMPK exhibited opposite changes. Furthermore, the survival rate of gastric cancer patients was positively correlated with p‐AMPK and negative with TGF‐β1. Therefore, our present studies depict a mechanism underlying AMPK suppression of TGF‐β1 autoinduction, which is mediated through inhibition of Smad3 phosphorylation and activation. Collectively, our study sheds a light on the potential usage of AMPK activators in the treatment of TGF‐β1‐mediated gastric cancer progression.

## 1 INTRODUCTION

Transforming growth factor‐β (TGF‐β) represents a superfamily consisting of 33 members of structurally related extracellular proteins, including the TGF‐β subfamily, bone morphogenetic proteins (BMPs), activins/inhibins, growth differentiation factors (GDFs), Müllerian inhibitory factor (MIF) and the glial cell line derived neurotrophic factor (GDNF).^1^ The TGF‐β signalling pathways regulate a variety of biological functions including cell growth, differentiation, organogenesis, angiogenesis, bone formation, immune regulation, fibrosis and tumorigenesis.^2^

Regardless of the diversity of TGF‐β ligands and their receptors, the intracellular signalling flow is similar. In general, TGF‐β ligands bind to specific pairs of receptor I and receptor II, thereby triggering phosphorylation of the receptor I on glycine‐serine‐rich (GS) region by the receptor II, despite that cross activation exists depending upon ligand concentration and cellular context.^3^ The activated receptor I phosphorylates canonical substrates, Smad2/3, or Smad1/5/8. On many circumstances, TGF‐β ligands also activate noncanonical pathways including ERK1/2, JNK, PI3K and Ras family small GTPases.^2^

TGF‐β signalling exerts dual effects on tumour development and progression. On the one hand, TGF‐β inhibits proliferation at early stages via blockade of the expression of cyclin dependent kinases (CDKs), leading to the attenuation of cell cycle progression.^4^ On the other hand, at late stages, TGF‐β enhances cancer progression and metastasis through promotion of epithelial to mesenchymal transition (EMT) and formation of cancer stem cells.^5^ Moreover, TGF‐β1 plays an important role in the development and maturation of Treg cells, which facilitates escaping of cancer cells from immune surveillance.^6^ In line with these findings, TGF‐β1 is highly expressed in advanced metastatic cancer. Thus, it serves as an indicator of cancer prognosis, therapeutic response and recurrence.^7^, ^8^, ^9^

Adenine monophosphate‐activated protein kinase (AMPK) is a protein kinase consisting of a catalytic subunit (including isoforms of α1, α2) and two regulatory subunits, β (β1, β2) and γ (γ1, γ2, γ3).^10^ As a cellular fuel gauge, it is activated under such stresses as hypoxia, glucose deprivation, ischaemia and reactive oxygen species. In these circumstances, AMP or AMP to ATP ratio is increased. AMP then binds to the γ subunits, inducing a conformational change that causes allosteric activation of AMPK, enables phosphorylation of Thr172 on the activation loop of the α subunit by upstream kinases such as LKB1 and prevents dephosphorylation of Thr172 by protein phosphatases, leading to a maximal activation of the enzyme.^11^, ^12^ Recently, another mechanism of AMPK activation independent of AMP was defined in response to glucose deprivation. The activation occurs in lysosomes where aldolase in the absence of fructose bis phosphates allows axin‐LKB1 complex to replace mTORC1 and phosphorylate AMPK.^13^

No matter what mechanism accounts for AMPK activation, the net effect is the increase of catabolism and attenuation of anabolism, resulting in increased production of ATP and preservation of energy for acute cell survival program. As AMPK increases glucose uptake into skeletal muscle, inhibits lipogenesis and enhances insulin sensitivity, it is well received a therapeutic target for metabolic syndrome and type 2 diabetes.^14^, ^15^ In fact, the first line anti‐diabetic drug metformin has been shown to be a pharmacological activator of AMPK. In the last decade, extensive studies have been carried out to delineate the role of AMPK in tumorigenesis and cancer progression.^16^ However, the conclusion is controversial. While some studies have shown that AMPK is activated in tumour microenvironment as a result of lacking nutrients and exerts a protective effect on tumorigenesis, a considerable number of studies have documented tumour suppressive function.^17^, ^18^, ^19^, ^20^

Previously, we and others have shown that activation of AMPK attenuates the TGF‐β1 signalling pathway via suppression of Smad2/3.^21^, ^22^, ^23^ Furthermore, we have reported that AMPK could reduce TGF‐β1 production in breast cancer,^24^ but the mechanism is not clear. The present study further explores the mechanism by which AMPK regulates TGF‐β1 expression and delineate correlation between AMPK and TGF‐β1 in gastric cancer.

## 2 MATERIALS AND METHODS

### 2.1 Reagents and chemicals

The gastric tissue micro‐array (TMA) was purchased from Shanghai Outdo Biotech Co. HRP‐conjugated secondary antibody and DAB kit was from ZSbio. Phenylmethylsulfonyl fluoride (PMSF), pepstatin A, metformin and berberine were from Sigma‐Aldrich. Aprotinin and Leupeptin were from GLPBio. EDTA and EGTA were from Solarbio. Human recombinant TGF‐β1 and monoclonal primary antibodies against β‐actin, p‐AMPKα Thr172, AMPKα, p‐Smad3 Ser423/425 and Smad3 were from Cell Signalling Technology. Monoclonal antibody against TGF‐β1 was from Abcam, and monoclonal antibody against LKB1 from Santa Cruz Biotechnology. Lipofectamine3000 was from Thermo Fisher Scientific Inc. FITC‐conjugated donkey anti‐rabbit antibody was from Life Technologies. RNAsimple Total RNA kit was from TIANGENE BioTec. Dual luciferase assay kit was from Promega Corporation. Restriction enzymes XhoI and HindIII and T4 ligase were from New England Biolabs. TGF‐β1 ELISA kit was purchased from Merck.

### 2.2 Human sample collections

The gastric TMA contained 98 cases of gastric carcinoma and paired adjacent noncancerous tissues aged from 32 to 84 years old (median age is 65 years old). All tissues were collected from patients after surgery by Shanghai Outdo Biotech Co from July 2006 to April 2007. The information on disease prognosis and survival length from May 2007 through June 2012 was archived and provided by the company.

The tumour and adjacent normal tissues were embedded in slides with a Φ1.5 mm spot. No patients received any chemotherapy or radiotherapy before surgery. Detailed information is listed in Supplementary Table 1. Tumour TNM staging were performed based on the 7th Edition of American Joint Committee on Cancer (AJCC) staging system. Histological grading was performed according to classification of tumours of the digestive system by the World Health Organization (WHO) in 2010.

Plasma were collected from patients with type 2 diabetes treated with metformin (21 males, 19 females) or with other glucose‐lowering drugs (24 males, 24 females) in the outpatient clinic of the First Affiliated Hospital, Nanchang University. Control samples were collected from healthy people who undertook annual health check in the hospital clinics. The ages of patients ranged from 45 to 65 years. Blood samples were obtained under the consent of patients.

The study on plasma levels of TGF‐β1 was approved by the Ethical Committee of the First Affiliated Hospital of Nanchang University (Ethical Trial for Medical Research, 2015(025)).

### 2.3 Immunohistochemistry

The assay was conducted as previously described by Huang et al^25^ Briefly, the section was sliced into 1.5 mm × 4 μm. The tissue slides were deparaffinized, rehydrated and then retrieved with citric acid buffer (pH 6.0, 10 mM) using standard microwave‐based method. The tissues were blocked, and then incubated with primary antibodies overnight. HRP‐conjugated secondary antibody was used and incubated at 37℃ for 30 minutes. DAB was used to develop the reaction. The slides were evaluated separately by 2 pathologists according to Germany semi‐quantitative method.^26^

The survival rate was estimated using the Kaplan‐Meier method.

### 2.4 Cell culture, transfection and drug treatment

Gastric cancer cells, MKN‐28 and SGC‐7901, were cultured in DMEM supplemented with 10% FBS at 5% CO_2_ and 37^0^c. SGC‐7901‐LKB1 cells were derived from SGC‐7901 cells by infection with lentivirus encoding LKB1, as described previously.^24^

For cell transfection, cells were incubated in Opti‐medium 4 hours, and then transfected with plasmids lipofectamine 3000 for 4‐6 hours. Cells were incubated with TGF‐β1 and/or metformin or berberine at doses and for different period of time as indicated in the text.

### 2.5 Measurement of TGF‐β1 levels

Patient's plasma were collected from patients with type 2 Diabetes from the Endocrinology Department and from healthy subjects for annual physical check from Physical Examination Center, the First Affiliated Hospital of Nanchang University. TGF‐β1 concentrations were measured with ELISA kit following the protocols provided by the manufacture.

For measurement of TGF‐ β1 secretion, SGC‐7901 and SGC‐7901‐LKB1 cells were cultured to 80% confluence. Cell culture media were replaced with DMEM plus 0.1% FBS and culture continued in the presence or absence of metformin or berberine for additional 8 hours. Cell culture media were collected and assayed on TGF‐ β1 using ELISA kit.

### 2.6 Western blot

The assay was conducted using standard method. In brief, cell lysates were prepared in RIPA buffer supplemented with protease inhibitors, consisting of PMSF (1 mM), aprotinin (2 µg/mL), leupeptin (5 µg/mL), pepstatin A (1 µg/mL), EDTA (5 mM) and EGTA (1 mM). Protein concentrations were measured by the Coomassie brilliant blue staining method. Cell lysates (10‐30 µg) were separated on SDS‐PDGE and transferred onto PVDF membranes. After blocking with 5% milk, the membranes were incubated with primary antibodies overnight at 4℃, as indicated in the figure legends, followed by incubation with HPR conjugated secondary antibodies. The blots were stained using ECL kit and visualized with ChemiDoc™ XRS + imaging system.

### 2.7 Dual luciferase assay

The promoter sequence was selected from GeneBank (NC_000019.10). A 1354bp fragment upstream of TGF‐β1 transcription start site was amplified from genomic DNA isolated from 293T cells by PCR and cloned to upstream of firefly luciferase in pGL3 plasmid at XhoI and HindIII. The insert was verified by sequencing. As indicated in the Results section, a series of deletion mutations (eg TGBP1, TGBP2, TGBP6, and TGBP7) and deletion mutation of a Smad3 binding site encompassing bp‐534~‐525 upstream of transcriptional start site (5′TGTCTGCCTC3′, complementary: 5′GAGGCAGACA3′) were made using PCR.

The pGL3 plasmids containing the TGF‐ β1 promoter variants were co‐transfected with Renilla luciferase plasmid into SGC‐7901‐LKB1 cells using Lipofectamine 3000. After 24 hours, the cells were treated with TGF‐β1 (5 ng/mL) and/or metformin (10 mM) for 8 hours. Luciferase activity was determined using a Dual luciferase assay kit and expressed as ratio of firefly luciferase to Renilla luciferase reading units.

### 2.8 RNA extraction and Quantitative real‐time PCR

Total RNA was extracted using the RNAsimple Total RNA kit following the manufacturer's instructions. Two micrograms of total RNA was used for cDNA synthesis. Real‐time PCR was carried out using SYBGREEN PCR kit with the PCR instrument (Applied Biosystems) following the manufacturer's instruction. Each sample was amplified in triplicates and normalized with glyceraldehyde 3‐phosphate dehydrogenase (GAPDH), which was then evaluated by the comparative threshold cycle value method (2 − ΔΔ C*_t_*) for relative quantification of gene expression.^22^

The primers for real‐time PCR were:

GAPDH: forward 5′‐ CATCTTCCAGGAGCGAGACC‐3′,

reverse 5′‐ CTCGTGGTTCACACCCATC‐3′;

TGF‐β1: forward 5′‐GGCCAGATCCTGTCCAAGC‐3′.

reverse 5′‐GTGGGTTTCCACCATTAGCAC‐3′.

### 2.9 Statically analysis

All the data were analysed using SPSS (Statistical Package for the Social Sciences) 20.0 (IBM Inc). Chi‐square test was used to compare the difference of IHC results and Spearman analysis (ranked data) or Pearson analysis (Continuous data) to examine the correlation between 2 proteins’ expression in the tissues. Quantitative data between groups were expressed as mean ± standard deviation (SD) and analysed by student *t*‐ test, or one‐way ANOVA for more than 2 groups. A *P* value equal to or less than .05 was statistically defined significance.

## 3 RESULTS

### 3.1 AMPK activation attenuates TGF‐β1 expression in human gastric cancer cell lines

**FIGURE 1:**
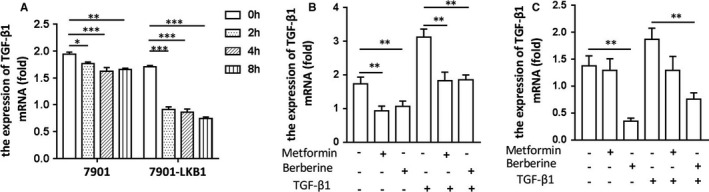
AMPK activation reduces mRNA abundance of TGF‐β1 in human gastric cancer cells. A, SGC‐7901 and SGC‐7901‐LKB1 cells were treated with metformin (10 mM) for different time and quantification of TGF‐β1 mRNA was conducted using qPCR. Relative expression was normalized with GAPDH. B, SGC‐7901‐LKB1 cells were treated with metformin (10 mM) or berberine (5 µM) for 4 hours, followed with or without TGF‐β1 (1 ng/mL) for an additional hour. TGF‐β1 mRNA was prepared and quantified as for A. C, MKN‐28 cells were treated with metformin or berberine, followed with or without TGF‐β1, and mRNA quantified as described for B. All assays were performed in triplicates ((mean ± SD, n = 3). One‐way ANOVA was used to assess significance. *P < .05, **P < .01. ***P < .001

Our previous study has shown that the plasma TGF‐β1 level is decreased in patients with type 2 diabetes after treatment with metformin, as compared to other glucose‐lowering drugs.^24^ As metformin is a well‐accepted pharmacological activator of AMPK, we examined if AMPK exerted an inhibitory effect on the expression of TGF‐β1 in cancer cells. First, we employed SGC‐7901 cells where LKB1 was barely detected, and constructed a stable cell line using lentivirus encoding LKB1.^25^ We then treated the cells with metformin or berberine, two AMPK activators, and performed qPCR Our result showed that both metformin (Figure 1A) and berberine (Figure 1B) significantly suppressed the level of TGF‐β1 with much greater effect in 7901‐LKB1 cells. The inhibition of TGF‐β1 in SGC‐7901‐LKB1 by metformin or berberine occurred under both basal and TGF‐β1‐treated conditions (Figure 1B). In parallel, we examined the effect of AMPK activators in MKN‐28 cells, another gastric cancer cell line (Figure 1C). The results showed similar inhibitory effects of metformin and berberine in this cell line.

**FIGURE 2:**
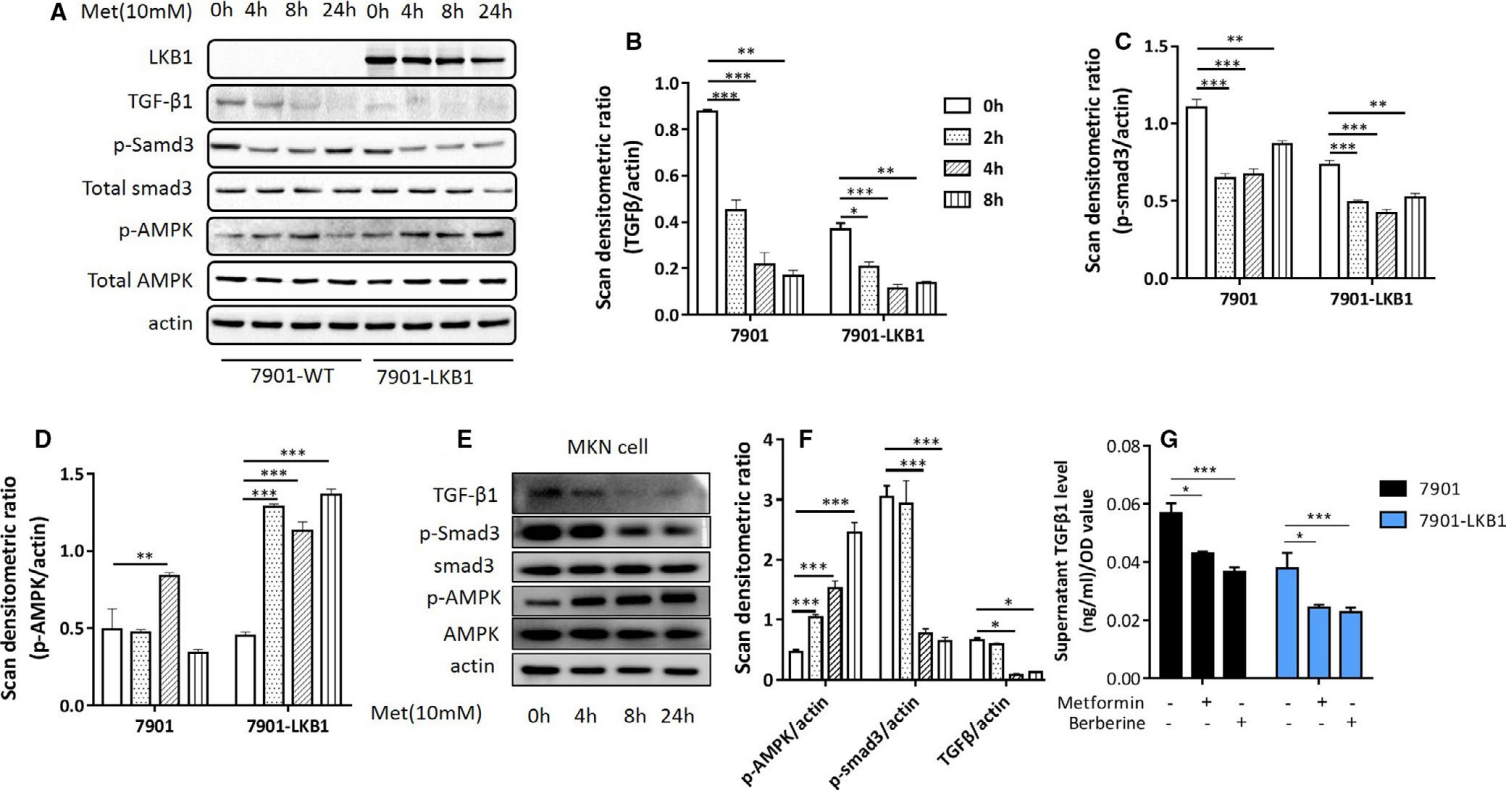
AMPK activation attenuates TGF‐β1 expression in human gastric cancer cells. A, SGC‐7901 and SGC‐7901‐LKB1 cells were treated with metformin (10 mM) for different time and cell extracts blotted with antibodies as indicated. A representative blot of three independent experiments is presented. B‐D, Western blots of A were scanned and densitometric unit ratio plotted. E, MKN‐28 cells were treated with metformin (10 mM) or berberine (5 µM) for 4 hours and cell extracts immunoblotted with antibodies as indicated. F, Western blots of E were scanned and densitometric unit ratio was plotted. G, SGC‐7901 and SGC‐7901‐LKB1 were treated with or without metformin or berberine for 8 hours and TGF‐β1 was measured in cell‐culture supernatant. All experiments were performed in triplicates or three times independently (mean ± SD). One‐way ANOVA was used to assess significance of difference, *P < .05, **P < .01, ***P < .001

Consistently, Western‐blot analysis revealed metformin suppressed protein expression TGF‐β1 (Figure 2A‐D). Likewise, metformin and berberine attenuated expression of TGF‐β1 in MKN‐28 cells (Figure 2D,E). We then assessed if metformin or berberine inhibited the release of TGF‐β1 from cells. Toward this end, SGC‐7901 and SGC‐7901‐LKB1 cells were switched to 0.1% FBS and cultured in the presence of absence or metformin or berberine. The results in Figure 2G showed that the release of TGF‐β1 was higher in SGC‐7901 cells than that of SGC‐7901‐LKB1 cells and both compounds reduced TGF‐β1 levels with greater effects in the SGC‐7901‐LKB1 cells. Altogether, these results strongly indicate the inhibition of TGF‐β1 expression by AMPK, possibly through transcriptional regulation.

### 3.2 AMPK inhibits the transcription of TGF‐β1 through Smad3

**FIGURE 3:**
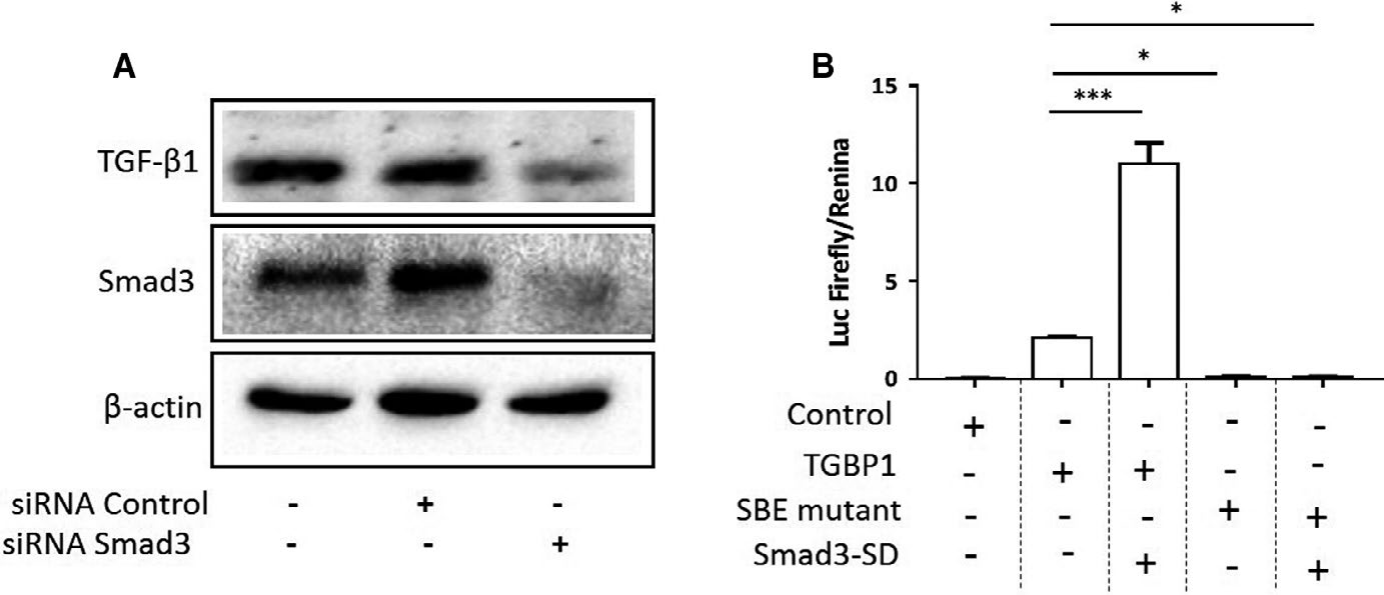
Smad3 acts on the enhancer on TGF‐β1 promoter. A, SGC‐7901‐LKB1 cells were transfected with Smad3 siRNA or scrambled siRNA as a control. Forty‐eight hours later, cell extracts were blotted with antibodies as indicated. B, The promoter fragment containing 1.35 kb 5′ of transcription start site (TGBP1) and the same fragment deleted of the putative Smad3 binding site (TGTCTGCCTC, SBE‐mutant) were cloned into the firefly luciferase reporter plasmid and co‐transfected with Renilla luciferase plasmid with or without the active mutant of Smad3 (Smad3‐SD) into SGC‐7901‐LKB1 cells. Luciferase activity in the cell lysates was measured and normalized to Renilla activity. The assays were performed in triplicates (mean ± SD). One‐way ANOVA was used to assess significance of differences between TGBP1 and tested groups. *P < .05, ***P < .001

We asked if AMPK inhibits the transcription of TGF‐β1 via the regulation of Smad3 as previous studies have shown that TGF‐β1 exerts forward feedback regulation of its expression.^27^ First, we tested if Smad3 played a role in the expression of TGF‐β1 in the present setting. Thus, SGC‐7901‐LKB1 cells were transfected with Smad3 siRNA or scrambled siRNA as a control. Western‐blot results showed a decrease in TGF‐β1 when Smad3 was downregulated (Figure 3A). Next, we ascertained if the regulation of TGF‐β1 expression was mediated by binding of Smad3 to the enhancer sequence on the promoter region. Toward this end, we cloned a genomic DNA fragment bearing 1.35 kb 5′ of the transcription initiation site and subcloned it to the promoterless firefly luciferase with or without deletion of the putative Smad3 binding element (−534 5′TGTCTGCCTC3′‐525, complementary: 5′GAGGCAGACA3′) (SBE‐mutant). We then transfected the wild type (TGBP1) or SBE‐mutant into SGC‐7901‐LKB1 cells. The firefly luciferase activity assay revealed that deletion of the SBE abolished the promoter activity (Figure 3B, column 2 vs column 4). Transfection of the active mutant of Smad3 (Smad‐3D) markedly enhanced the luciferase activity, whereas SBE‐mutant was unresponsive. These results demonstrated that Smad3 enhanced transcription of TGF‐β1.

**FIGURE 4:**
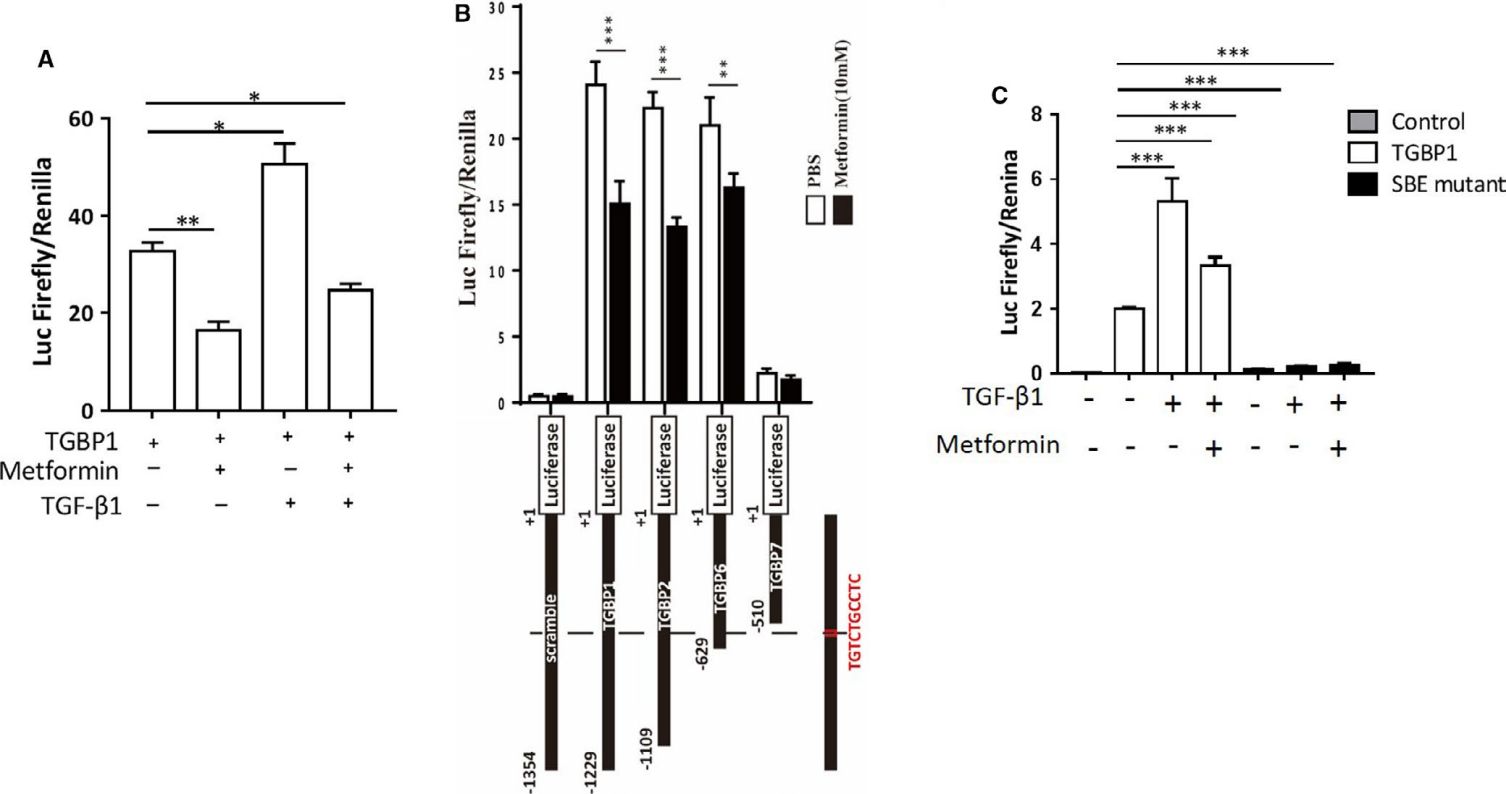
Metformin suppresses the TGF‐β1 promoter activity via regulation of Smad3. A, TGFPP1 firefly luciferase plasmid and the Renilla luciferase plasmid were co‐transfected into SGC‐7901‐LKB1 cells. Forty‐eight hours later, the cells were treated with or without metformin (10 mM) for 8 hours. Luciferase activity assay was performed as for Figure 3B. B, The promoter fragment containing 1.35 kb 5’ of transcription initiation site was truncated from the 5’ end to different sizes. Different fragments or SBE‐mutant linked with firefly luciferase were co‐transfected with Renilla luciferase into SGC‐7901‐LKB1 cells treated with/without metformin (10 mM) for 8 hours. C, TGBP1 or SBE‐mutant was co‐transfected with Renilla luciferase. All experiments were conducted in triplicates (mean ± SD). One‐way ANOVA was used to assess significance of difference. *P < .05, ***P < .001

In next experiment, we assessed if metformin suppressed transcription of TGF‐β1. Thus, TGBP1‐luciferase plasmids were transfected into SGC‐7901‐LKB1 cells. Forty‐eight hours later, the cells were treated with TGF‐β1 and/or metformin for 8 hours. The results revealed that metformin reduced luciferase activity on both basal and TGF‐β1‐stimulated conditions (Figure 4A). We then transfected a series of truncation mutants of the TGF‐β1 promoter region into the cells. As shown in Figure 4B, when the truncation was made down to −629 bp, the promoter activity was kept well and significantly suppressed by metformin. However, deletion to −510 bp passing the SBE abolished the promoter activity. Without SBE, the promoter was not responsive to TGF‐β1 and metformin (Figure 4C). Altogether, our data showed that metformin via activation of AMPK inhibited TGF‐β1‐induced activation of Smad3, leading to suppression of autoinduction of TGF‐β1.

### 3.3 Metformin reduces plasma TGF‐β1 levels

**FIGURE 5:**
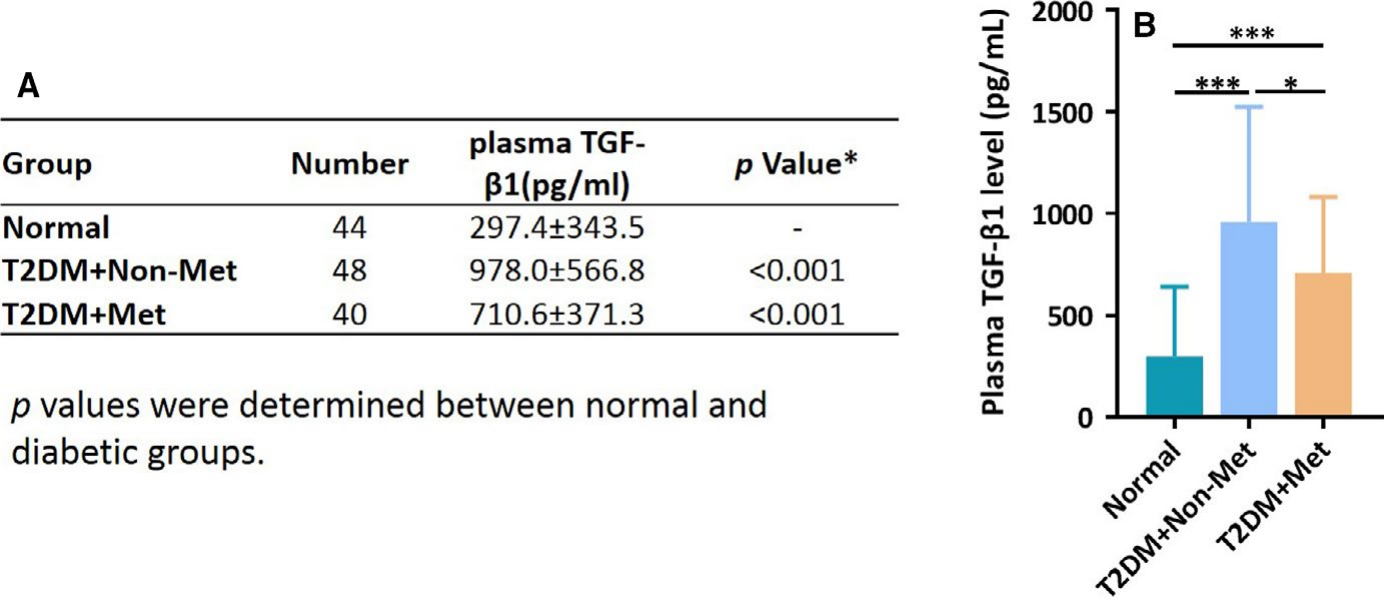
Metformin reduces plasma levels of TGF‐β1 in type 2 diabetic patients. A, Plasma levels of TGF‐β1 were measured in the type 2 diabetic patients treated with metformin or other glucose‐lowering drugs, as compared to normal subjects. Data including sample number and values are presented in Table. B, Bar graph represents the levels of plasma TGF‐β1 (Mean ± SD). One‐way ANOVA was used to assess significance of difference, *P < .05, ***P < .001

Our previous study showed that serum level of TGF‐β1 was reduced in type 2 diabetes patients treated with metformin, where health subjects were not recruited as a control.^24^ In the present study, we included healthy people and expanded the number of samples. As shown in Figure 5, our result revealed that plasma levels of TGF‐β1 were much greater in type 2 diabetes treated with non‐metformin drugs than that of healthy subjects (*P* < .001). Treatment with metformin significantly lowered TGF‐β1 level (*P* < .05). Although this result was not directly obtained from gastric cancer patients, it provided us clinical evidence in support of our in vitro data that metformin suppressed TGF‐β1 production.

### 3.4 TGF‐β1 is decreased in gastric cancer and correlated with AMPK activation

**FIGURE 6:**
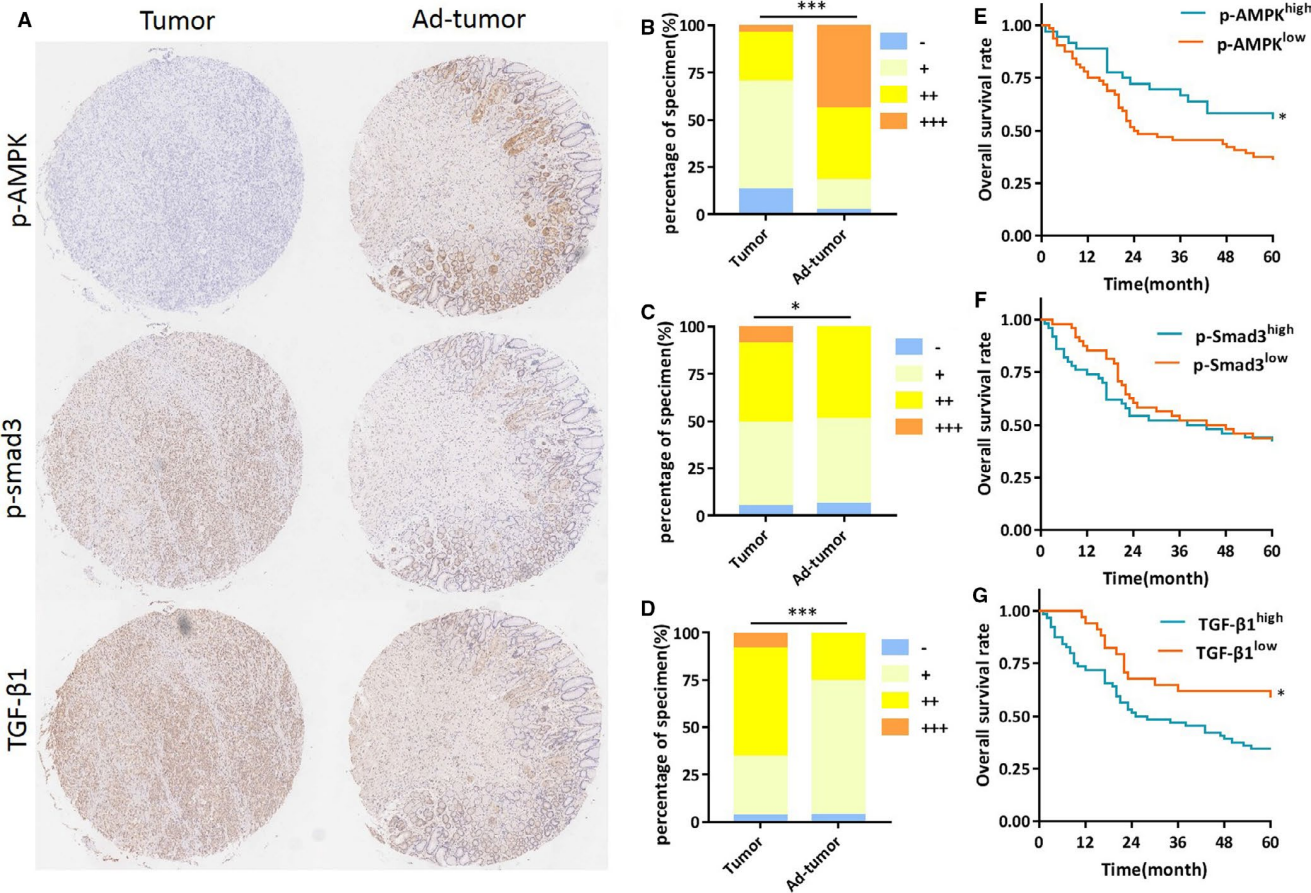
TGF‐β1 level is decreased in gastric cancer and correlated with AMPK activation. A, Representative images of p‐AMPK (Th172), p‐Smad3 (Ser423/425) and TGF‐β1 in gastric carcinoma and adjacent tissues. B‐D, Statistical results of p‐AMPK (Th172), p‐Smad3 (Ser423/425) and TGF‐β1 were evaluated, respectively, according to Germany semi‐quantitative method and plotted. Evaluation of immunochemistry data showed that p‐AMPK was less than adjacent tumour tissues (*P* < .001), while p‐Smad3 and TGF‐β1 were higher in tumour tissues (*P* < .05 and *P* < .001 respectively). E‐G, Kaplan‐Meier survival curve for 5 year overall survival (OS) was assessed as opposed to on protein expression levels. OS for patients with higher p‐AMPK level was 55.6%, compared to 35.9% with lower p‐AMPK level (*P* < .05). OS for patients with higher TGF‐β1 level was 34.9%, compared to 51.4% of lower TGF‐β1 level (*P* < .05). No significance of OS was found with p‐Smad3 (*P* > .05)

We assessed if there was correlation between TGF‐β1 expression and activation state of AMPK in gastric cancer. Thus, immunohistochemistry was performed on 98 paraffin embedded gastric cancer specimens, and p‐AMPK, p‐Smad3 and TGF‐β1 levels were examined. The patients’ information was presented in Supplementary Table 1. Representative images of immunohistochemical staining results are shown in Figure 6A. The expression level of p‐AMPK, p‐Smad3 and TGF‐β1 were evaluated. The results showed that p‐AMPK was expressed higher in adjacent normal tissues, as compared to tumour tissues (Chi square, ***P < .001, Figure 6B). In contrast, tumour tissue showed increased staining of p‐Smad3 (Chi square, *P < .05, Figure 6C) and TGF‐β1 (Chi square, **P < .01, Figure 6D). The spearman correlation test indicated that the expression levels of p‐AMPK and TGF‐β1 in the tumour was inversely correlated (No correlation with p‐Smad3 in this study, *R* ^2^ = 0.361, *P* < .01).

Next, we assessed the correlation of these three parameters with survival rate using Kaplan‐Meier plot. Our results showed that the survival rate of the patients positively correlated with p‐AMPK level in gastric cancer patients (Figure 6E) and negatively with the level of TGF‐β1 (Figure 6G). However, no significant correlation was found between p‐Smad3 level and the overall survival rate (Figure 6F).

## 4 DISCUSSION

Increased paracrine production of TGF‐β in cancer microenvironment contributes to cancer progression. Hence, targeting the production of TGF‐β represents an important approach for combating cancer progression. Our previous study has indicated that AMPK regulates TGF‐β1 production in breast cancer cells.^24^ The present study further delineated the underlying mechanism. Our results showed that metformin diminished TGF‐β1 at both mRNA and protein levels in gastric cancer cells, an event that was dependent on the presence of AMPK. We employed the luciferase reporter to characterize the promoter activity and found that metformin was able to inhibit the transcription of the reporter luciferase. We then identified a typical Smad3 binding site (GTCTG) in the promoter region of TGF‐β1 and found that deletion of this site abolished the promoter activity and response to metformin as well. In accordance, TGF‐β1 was highly expressed in human gastric cancer tissues as compared to adjacent normal tissues. In contrast, p‐AMPK exhibited opposite changes. Furthermore, the survival rate of gastric cancer patients was positively correlated with p‐AMPK and negative with TGF‐β1. Altogether, our results demonstrate that metformin suppresses autoinduction of TGF‐β1, and thus suggest that metformin could be used as a candidate in treatment of cancer progression.

Three isoforms of TGF‐β (TGF‐β1, TGF‐β2 and TGF‐β3) have been reported and are encoded by distinct genes.^28^, ^29^ Therein, the expression of TGF‐β1 and TGF‐β3 are modulated by auto‐feedback control loop.^27^ The early studies have shown that km23‐ 1, a TGF‐β receptor binding protein, serves as a critical adaptor coupling activation of the receptor to the Ras effector pathway required for autoregulation of TGF‐β1 production independent of Smad2.^30^ A second study demonstrated that *Clostridium butyricum,* a gram‐positive probiotic bacterial strain, potently induces production of TGF‐β1 from lamina propria dendritic cells (LPDCs) that recruits Toll‐like receptor 2 (TLR2) involving both Smad3 and ERK‐AP‐1 pathways.^27^ Interestingly, Smad3 signal is necessary for robust TGF‐β expression in LPDCs, whereas it is negatively regulated by Smad2. The study employed chromosome immunoprecipitation and luciferase reporter techniques to map the binding sites within bp‐196 to −113 upstream of the transcriptional initiation site, which contained core motifs (5′AGAC′). In the present study, we performed a stringent search on 1354 bp of the promoter region of TGF‐β and only identified one site (bp‐534~‐525 5′TGTCTGCCTC3′, complementary: 5′GAGGCAGACA3′). Our results showed that deletion beyond this site or site‐directed deletion of this site abolished promoter activity. We noticed that all these studies including ours render different mechanisms regardless the same outcomes. It is not clear at the reason underlying the discrepancy. It is possible that different cell contexts determining cellular responses to stimuli accounts for the difference.

It has been reported that AMPK regulates TGF‐β1 expression in various cell types. For example, Zhang et.al^31^ have shown that metformin could attenuate TGF‐β1 expression via hepatocyte nuclear factor‐4‐α in mouse cardiac fibroblasts. Studies of Xiao et.al^32^ have reported that metformin significantly reduces TGF‐β1 production in unilateral ureteral obstruction (UUO)‐induced renal fibrosis, which is mediated by activation of AMPK‐α2. Our current study has deciphered the mechanism by which metformin downregulates TGF‐β1 level. We have identified that metformin via activation of LKB1/AMPK inhibits TGF‐β1‐induced activation of Smad3 and eventually prevents binding of p‐Smad3 to the promoter of TGF‐β1, leading to attenuation of its production.

We have previously reported that metformin reduces the level of serum TGF‐β1 in patients with type 2 diabetes receiving metformin. However, the reduction was insignificant due to insufficient number of samples. Thus, our present study expanded the number of samples and included normal subjects as a control. The results revealed significant reduction of plasma TGF‐β1 by metformin treatment. Although the definitive answer as to whether metformin inhibits autoinduction of TGF‐β1 in human gastric cancer warrants vigorous tests upon approval by the ethical committee, the current data are in line with our in vitro findings. We should point out that the values of blood concentrations of TGF‐β1 varied between our present study and a previous publication.^24^ Several reasons might be attributed to the difference. For example, our first report measured serum levels of TGF‐β1, while the present study used plasma samples. Additionally, different assay kits might contribute to the variations. Indeed, we reviewed several publications on blood levels of TGF‐β1 and found the values are quite different from various studies.^33^, ^34^, ^35^ Nevertheless, our take home message is that metformin could reduce the blood TGF‐β1 level.

In conclusion, our work has demonstrated that activation of AMPK could lead to the inhibition of autoinduction of TGF‐β1, an important contributing factor for cancer progression. Furthermore, we firstly identified a canonical Smad3 binding sequence (5′‐TGTCTGCCTC‐3′) in the promoter region of TGF‐β1, that is the target site for metformin. This finding is especially meaningful, inasmuch as it points to a possibility that metformin as well as other clinically used AMPK activators could be used in the treatment or prevention of gastric cancer progression.

## CONFLICT OF INTEREST

We hereby declare that all co‐authors have no conflicts of interest in this work, including affiliations, financial relationships, personal relationships, or funding sources that could be perceived as influencing an author's objectivity regarding the manuscript content.

## AUTHOR CONTRIBUTION

**Junrong Zou:** Conceptualization (equal); Data curation (lead); Investigation (equal); Writing‐original draft (equal); Writing‐review & editing (equal). **Cong Li:** Investigation (equal). **Shanshan Jiang:** Data curation (equal); Investigation (equal); Methodology (equal). **Lingyu Luo:** Resources (equal); Writing‐review & editing (equal). **Xiaohua Yan:** Methodology (equal); Resources (equal). **Deqiang Huang:** Conceptualization (equal); Funding acquisition (equal); Resources (equal); Writing‐review & editing (equal). **Zhijun Luo:** Conceptualization (equal); Funding acquisition (lead); Methodology (lead); Project administration (supporting); Resources (lead); Writing‐original draft (equal); Writing‐review & editing (equal).

## Supporting information

Supplemental Table 1

## Data Availability

The data that supports the findings of this study are available in the supplementary material of this article.
