# Supplementary material for: AMPK inhibits Smad3‐mediated autoinduction of TGF‐β1 in gastric cancer cells
Source: J Cell Mol Med. 2021 Feb 3;25(6):2806–15. doi: 10.1111/jcmm.16308 (PMC7957200; doi:10.1111/jcmm.16308)
Supplement: Supplementary file 1 — Supplemental Table 1 [file JCMM-25-2806-s001.docx]

**Supplementary Table 1: The patients information in the gastric tumor tissue microarray (TMA)**

| **Tissue Code** | **Gender** | **Age** | **Histological type** | **Histological stage** | **T** | **N** | **M** | **TNM stage** | **Tumor size（cm^3^）** | **Tumor position** | **Survival months** |
| --- | --- | --- | --- | --- | --- | --- | --- | --- | --- | --- | --- |
| RDgStm0609A0579 | male | 61 | mucinous adenocarcinoma | Ⅲ | T3 | N2 | M0 | 3C | 4×3×1 | paries posterior ventriculi | 24 |
| RDgStm0609A0580 | male | 52 | adenocarcinoma, partial signet-ring cell carcinoma | Ⅲ | T3-4 | N3a | M0 | 3 | 7×6×2 | Antrum of stomach | 6 |
| RDgStm0609A0586 | male | 54 | adenocarcinoma | Ⅱ-Ⅲ | T3 | N3a | M0 | 3B | 3×2.5×0.5 | Antrum of stomach | 9 |
| RDgStm0609A0589 | male | 57 | adenocarcinoma | Ⅲ | T4b | N2 | M0 | 3C | 6.5×4.5×1 | curvature of the stomach | 9 |
| RDgStm0609A0590 | male | 77 | adenocarcinoma | Ⅲ | T3 | N3a | M1 | 4 | 7×4×1.5 | curvature of the stomach | 8 |
| RDgStm0609A0591 | male | 59 | adenocarcinoma | Ⅲ | T4b | N1 | M1 | 4 | 7×5.5×1 | Antrum of stomach | 12 |
| RDgStm0609A0593 | male | 84 | canalicular adenoma | Ⅱ-Ⅲ | T4a | N1 | M0 | 3A | 9×6.5×2 | Antrum of stomach | 22 |
| RDgStm0609A0594 | male | 77 | adenocarcinoma | Ⅱ | T2 | N2 | M0 | 2B | 3×2×0.5 | Gastric cardia | 16 |
| RDgStm0609A0595 | female | 69 | undifferentiated carcinoma, partial adenocarcinoma | Ⅲ-Ⅳ | T3 | N2 | M0 | 3A | 6×2.5×1.5 | Antrum of stomach | 19 |
| RDgStm0609A0597 | male | 73 | canalicular adenoma | Ⅱ-Ⅲ | T2 | N0 | M0 | 1B | 2×1.5×0.4 | Gastric cardia | 55 |
| RDgStm0609A0598 | female | 62 | canalicular adenoma | Ⅱ | T1 | N0 | M0 | 1A | 3×2.8×1 | Antrum of stomach | 23 |
| RDgStm0609A0601 | female | 75 | mucinous adenocarcinoma | Ⅲ | T2-3 | N1 | M0 | 2 | 1.8×1.5×1.2 | gastric body | 8 |
| RDgStm0609A0603 | male | 71 | adenocarcinoma | Ⅲ | T3-4 | N3a | M0 | 3 | 7.5×5×1 | Gastric cardia | 20 |
| RDgStm0609A0605 | male | 41 | undifferentiated carcinoma, partial adenocarcinoma | Ⅲ-Ⅳ | T4a | N3a | M0 | 3C | 6.5×6×1 | Antrum of stomach | 50 |
| RDgStm0609A0607 | female | 67 | canalicular adenoma | Ⅲ | T3 | N3a | M0 | 3B | 2.5×2.5×1 | Antrum of stomach | 4 |
| RDgStm0609A0610 | male | 67 | canalicular adenoma | Ⅱ-Ⅲ | T3 | N3a | M0 | 3B | 15×8×1.5 | gastric body | 22 |
| RDgStm0609A0622 | male | 60 | adenocarcinoma | Ⅲ | T3 | N2 | M0 | 3A | 12×9×1.5 | gastric body | 12 |
| RDgStm0611A0631 | female | 56 | undifferentiated carcinoma, partial adenocarcinoma | Ⅲ-Ⅳ | T3 | N2 | M0 | 3A | 18×11×1 | whole stomach | 23 |
| RDgStm0611A0634 | female | 53 | adenocarcinoma | Ⅱ | T4a | N3a | M0 | 3C | 8×7×1.5 | Gastric cardia | 17 |
| RDgStm0611A0635 | male | 53 | adenocarcinoma | Ⅱ | T4a | N0 | M0 | 2B | 3.5×3.5×1.5 | Antrum of stomach | 3 |
| RDgStm0611A0636 | female | 73 | adenocarcinoma | Ⅲ | T2 | N3a | M0 | 3A | 3×2×1 | curvature of the stomach | 20 |
| RDgStm0701A0829 | female | 65 | canalicular adenoma | Ⅲ | T3 | N2 | M0 | 3A | 5×4.8×0.2 | Antrum of stomach | 6 |
| RDgStm0701A0830 | male | 71 | canalicular adenoma | Ⅱ-Ⅲ | T3 | N1 | M0 | 2B | 4.5×5×1.5 | Gastric cardia | 15 |
| RDgStm0701A0833 | male | 48 | canalicular adenoma | Ⅱ-Ⅲ | T3 | N2 | M0 | 3A | 8.5×7×5 | gastric angle | 22 |
| RDgStm0701A0834 | male | 72 | adenocarcinoma | Ⅲ | T3 | N3a | M0 | 3B | 7×5×4 | Antrum of stomach | 3 |
| RDgStm0704A0896 | male | 70 | adenocarcinoma | Ⅲ | T2 | N0 | M0 | 1B | 8×7×6 | Antrum of stomach | 48 |
| RDgStm0704A0900 | female | 77 | adenocarcinoma | Ⅲ | T3 | N3a | M0 | 3B | 6×6×5 | whole stomach | 53 |
| RDgStm0704A0901 | male | 75 | adenocarcinoma | Ⅲ | T3 | N0 | M0 | 2A | 4×2×1.5 | gastric angle | 34 |
| RDgStm0704A0905 | male | 72 | adenocarcinoma | Ⅱ | T3 | N2 | M0 | 3A | 15×13×11 | Gastric cardia | 60 |
| RDgStm0704A0914 | male | 79 | undifferentiated carcinoma, partial adenocarcinoma | Ⅲ-Ⅳ | T3 | N0 | M0 | 2A | 10×10×8.2 | Gastric cardia | 11 |
| RDgStm0704A0916 | female | 63 | adenocarcinoma | Ⅱ | T3 | N0 | M0 | 2A | 3×2×1.5 | pylorus | 17 |
| RDgStm0704A0918 | male | 65 | adenocarcinoma, partial mucinous adenocarcinoma | Ⅱ-Ⅲ | T3 | N1 | M0 | 2B | 1.2-2×1.5×1.5 | gastric body | 47 |
| RDgStm0704A0962 | female | 68 | mucinous adenocarcinoma | Ⅲ | T3 | N3a | M0 | 3B | 7×6×1.5 | Antrum of stomach | 21 |
| RDgStm0704A0919 | male | 78 | adenocarcinoma | Ⅱ | T3 | N2 | M0 | 3A | 3.5×3×2 | Antrum of stomach | 10 |
| RDgStm0704A0920 | female | 57 | adenocarcinoma | Ⅱ-Ⅲ | T3 | N3a | M1 | 4 | 7×7×4 | Antrum of stomach | 20 |
| RDgStm0704A0921 | male | 45 | adenocarcinoma | Ⅱ | T3 | N2 | M1 | 4 | 4×3×1.5 | Antrum of stomach | 4 |
| RDgStm0704A0923 | female | 50 | adenocarcinoma | Ⅲ | T3 | N2 | M0 | 3A | 2.5×2×1 | gastric angle | 20 |
| RDgStm0704A0927 | male | 65 | canalicular adenoma | Ⅲ | T2-3 | N0 | M0 | 2 | 4×3.5×1 | Antrum of stomach | 25 |
| RDgStm0704A0928 | male | 71 | canalicular adenoma | Ⅱ-Ⅲ | T3 | N2 | M1 | 4 | 3.5×3×0.4 | Antrum of stomach | 30 |
| RDgStm0704A0933 | female | 75 | adenocarcinoma | Ⅲ | T3-4 | N1 | M0 | 2B | 3×2.5×1.7 | Antrum of stomach | 3 |
| RDgStm0704A0934 | male | 32 | undifferentiated carcinoma, partial adenocarcinoma | Ⅲ-Ⅳ |  | N3a | M1 | 4 | 2.0×7×1.5 | whole stomach | 2 |
| RDgStm0704A0935 | male | 58 | adenocarcinoma | Ⅱ-Ⅲ | T3 | N0 | M0 | 2A | 8×7×6 | gastric body | 60 |
| RDgStm0704A0936 | female | 73 | undifferentiated carcinoma, partial adenocarcinoma | Ⅲ-Ⅳ | T3 | N2 | M0 | 3A | 14×10×7 | curvature of the stomach | 60 |
| RDgStm0704A0938 | female | 62 | undifferentiated carcinoma, partial adenocarcinoma | Ⅲ-Ⅳ | T3 | N0 | M1 | 4 | 12×8×1 | Antrum of stomach | 60 |
| RDgStm0704A0939 | female | 73 | canalicular adenoma | Ⅱ-Ⅲ | T4a | N2 | M0 | 3B | 4×3×2 | stomach | 60 |
| RDgStm0704A0941 | female | 58 | canalicular adenoma | Ⅱ | T3 | N1 | M0 | 2B | 4×2×1 | Antrum of stomach | 60 |
| RDgStm0704A0942 | male | 63 | mucinous adenocarcinoma | Ⅲ | T3 | N3a | M0 | 3B | 5×4×1 | Gastric cardia | 60 |
| RDgStm0704A0944 | male | 59 | adenocarcinoma, partialsignet-ring cell carcinoma | Ⅲ | T2 | N0 | M0 | 1B | 3×2×1 | lesser curvature | 60 |
| RDgStm0704A0947 | male | 72 | signet-ring cell carcinoma, partial mucinous adenocarcinoma | Ⅲ | T3 | N0 | M0 | 2A | 5×2.5×1 | Antrum of stomach | 60 |
| RDgStm0704A0949 | male | 62 | adenocarcinoma | Ⅱ | T1b | N0 | M0 | 1A | 4.5×2.5×0.3 | Gastric cardia | 60 |
| RDgStm0704A0950 | female | 73 | adenocarcinoma | Ⅲ | T3 | N2 | M0 | 3A | 1-4×3×3 | whole stomach | 60 |
| RDgStm0704A0952 | male | 52 | canalicular adenoma | Ⅲ | T3 | N3b | M0 | 3B | 15×10×10 | lesser curvature | 60 |
| RDgStm0704A0953 | male | 50 | undifferentiated carcinoma, partial adenocarcinoma | Ⅲ-Ⅳ | T3 | N3a | M0 | 3B | 6×6×5 | Antrum of stomach | 60 |
| RDgStm0704A0954 | female | 74 | canalicular adenoma | Ⅱ-Ⅲ | T3 | N3a | M1 | 4 | 5×3×1 | Antrum of stomach | 60 |
| RDgStm0704A0956 | female | 67 | signet-ring cell carcinoma | Ⅲ | T4b | N3a | M0 | 3C | 10×8×2 | Antrum of stomach | 60 |
| RDgStm0704A0957 | male | 53 | mucinous adenocarcinoma | Ⅲ | T3 | N2 | M0 | 3A | 5.5×4.5×3.5 | Antrum of stomach | 60 |
| RDgStm0704A0958 | male | 65 | adenocarcinoma | Ⅱ | T3 | N1 | M0 | 2B | 0.5×1.2×0.6 | lesser curvature | 60 |
| RDgStm0704A0963 | female | 52 | signet-ring cell carcinoma | Ⅲ | T3 | N3a | M0 | 3B | 5×4×1 | gastric body | 60 |
| RDgStm0705A1052 | male | 47 | adenocarcinoma | Ⅲ | T4a | N3a | M1 | 4 | 6×5×1.3 | Antrum of stomach | 60 |
| RDgStm0705A1055 | male | 62 | canalicular adenoma | Ⅱ-Ⅲ | T2-3 | N1 | M0 | 2 | 3.5×2.5×0.5 | Gastric cardia | 60 |
| RDgStm0705A1056 | male | 66 | adenocarcinoma, partial signet-ring cell carcinoma | Ⅲ | T1b | N2 | M0 | 2A | 6×5.5×0.3 | Antrum of stomach | 60 |
| RDgStm0705A1057 | female | 67 | canalicular adenoma | Ⅱ-Ⅲ | T3 | N0 | M0 | 2A | 3.5×3×1 | Antrum of stomach | 60 |
| RDgStm0705A1058 | female | 59 | adenocarcinoma, partial signet-ring cell carcinoma | Ⅱ-Ⅲ | T1a | N1 | M0 | 1B | 3×2.1×0.4 | whole stomach | 60 |
| RDgStm0705A1059 | female | 74 | adenocarcinoma | Ⅱ | T3 | N2 | M0 | 3A | 5×3×1 | Antrum of stomach | 60 |
| RDgStm0705A1060 | female | 58 | adenocarcinoma, partial signet-ring cell carcinoma | Ⅲ | T3 | N3a | M0 | 3B | 5×4×1 | gastric angle | 36 |
| RDgStm0705A1061 | male | 77 | mucinous adenocarcinoma | Ⅲ | T3 | N1 | M0 | 2B | 6×2.5×1.5 | Antrum of stomach | 60 |
| RDgStm0705A1062 | female | 57 | adenocarcinoma | Ⅱ | T1b | N1 | M0 | 1B | 3×2×0.6 | lesser curvature | 60 |
| RDgStm0705A1064 | male | 78 | adenocarcinoma | Ⅲ | T3 | N3a | M0 | 3B | - | lesser curvature | 1 |
| RDgStm0705A1066 | male | 76 | mucinous adenocarcinoma | Ⅲ | T3 | N1 | M0 | 2B | 7.5×7.5×2 | lesser curvature | 17 |
| RDgStm0705A1067 | male | 56 | adenocarcinoma | Ⅱ | T3 | N2 | M0 | 3A | - | whole stomach | 60 |
| RDgStm0705A1068 | male | 51 | adenocarcinoma | Ⅲ | T3 | N2 | M0 | 3A | 7×4×1 | Antrum of stomach | 17 |
| RDgStm0705A1070 | female | 57 | undifferentiated carcinoma, partial adenocarcinoma | Ⅲ-Ⅳ | T3 | N0 | M0 | 2A | 9×5×1 | gastric body | 60 |
| RDgStm0705A1072 | male | 52 | adenocarcinoma | Ⅲ | T3 | N2 | M0 | 3A | 3×3×1 | Antrum of stomach | 17 |
| RDgStm0705A1074 | male | 61 | adenocarcinoma | Ⅱ-Ⅲ | T3 | N2 | M0 | 3A | 5×4×1 | Antrum of stomach | 60 |
| RDgStm0705A1076 | female | 67 | undifferentiated carcinoma, partial adenocarcinoma | Ⅲ-Ⅳ | T2-3 | N0 | M0 | 2 | 3.5×3×1 | lesser curvature | 17 |
| RDgStm0705A1078 | male | 62 | adenocarcinoma | Ⅲ | T3 | N3a | M0 | 3B | 5×5×1 | gastric body | 60 |
| RDgStm0705A1080 | male | 74 | adenocarcinoma | Ⅱ-Ⅲ | T4a | N0 | M0 | 2B | 4×2×1 | gastric body | 9 |
| RDgStm0705A1083 | female | 81 | adenocarcinoma | Ⅲ | T3 | N0 | M0 | 2A | 2.8×2.5×1 | Antrum of stomach | 60 |
| RDgStm0705A1085 | male | 62 | adenocarcinoma | Ⅲ | T1b | N0 | M0 | 1A | 1.8×1.7×0.2 | Antrum of stomach | 28 |
| RDgStm0705A1086 | male | 62 | adenocarcinoma | Ⅲ | T4a | N3b | M0 | 3C | 5.5×3.5×1.5 | gastric angle | 7 |
| RDgStm0706A1162 | female | 77 | adenocarcinoma | Ⅱ-Ⅲ | T3 | N0 | M0 | 2A | 10×7.5×1 | Antrum of stomach | 38 |
| RDgStm0706A1163 | male | 61 | adenocarcinoma | Ⅲ | T2 | N3a | M0 | 3A | 6.5×5.5×1 | Antrum of stomach | 60 |
| RDgStm0609A0578 | male | 79 | signet-ring cell carcinoma | Ⅲ | T4a | 有 | M0 | 3 | 4.5×3×1 | Antrum of stomach | 60 |
| RDgStm0609A0587 | male | 48 | adenocarcinoma, partial signet-ring cell carcinoma | Ⅲ | T3 | N0 | M0 | 2A | 2.5×2.3×1.5 | gastric body小 | 60 |
| RDgStm0609A0606 | female | 69 | mucinous adenocarcinoma | Ⅲ | T3 | N0 | M0 | 2A | 6×5.5×1 | Antrum of stomach | 60 |
| RDgStm0609A0611 | male | 65 | canalicular adenoma | Ⅱ-Ⅲ | T3 | N0 | M0 | 2A | 3×2×1.5 | Antrum of stomach | 43 |
| RDgStm0611A0632 | male | 79 | canalicular adenoma | Ⅲ | T4a | N3a | M0 | 3C | 7.5×4.5×1.5 | Antrum of stomach | 43 |
| RDgStm0704A0895 | male | 53 | adenocarcinoma | Ⅲ | T3 | N0 | M0 | 2A | 6×6×3 | Antrum of stomach | 60 |
| RDgStm0704A0898 | male | 66 | adenocarcinoma | Ⅲ | T3-4 | N2 | M0 | 3 | 15×13×11 | Antrum of stomach | 60 |
| RDgStm0704A0910 | male | 49 | undifferentiated carcinoma, partial adenocarcinoma | Ⅲ-Ⅳ | T3 | N3b | M0 | 3B | 20×13×1 | whole stomach | 21 |
| RDgStm0704A0911 | male | 73 | adenocarcinoma | Ⅲ | T4a | N3b | M0 | 3C | 14×10×7 | Antrum of stomach | 4 |
| RDgStm0704A0937 | male | 70 | adenocarcinoma | Ⅱ-Ⅲ | T2 | N0 | M0 | 1B | 6×6×3 | Gastric cardia | 23 |
| RDgStm0704A0945 | female | 54 | adenocarcinoma | Ⅲ | T3 | N0 | M0 | 2A | 7×4×1.5 | Antrum of stomach | 60 |
| RDgStm0704A0951 | male | 68 | adenocarcinoma | Ⅲ | T3 | N2 | M0 | 3A | 3.5×2×2 | lesser curvature | 60 |
| RDgStm0704A0955 | female | 55 | signet-ring cell carcinoma | Ⅲ | T3 | N1 | M0 | 2B | 4×3×1 | gastric body | 60 |
| RDgStm0705A1051 | female | 65 | undifferentiated carcinoma, partial adenocarcinoma | Ⅲ-Ⅳ | T3 | N3a | M0 | 3B | 7×6×2 | Antrum of stomach | 60 |
| RDgStm0705A1054 | male | 72 | canalicular adenoma | Ⅱ-Ⅲ | T4a | N1 | M0 | 3A | 4.5×3.5×1.2 | Antrum of stomach | 60 |
| RDgStm0705A1084 | female | 81 | adenocarcinoma | Ⅲ | T4a | N1 | M0 | 3A | 5×3×1.2 | Antrum of stomach | 60 |
